# The Pediatric Obsessive-Compulsive Disorder Treatment Study II: rationale, design and methods

**DOI:** 10.1186/1753-2000-3-4

**Published:** 2009-01-30

**Authors:** Jennifer B Freeman, Molly L Choate-Summers, Abbe M Garcia, Phoebe S Moore, Jeffrey J Sapyta, Muniya S Khanna, John S March, Edna B Foa, Martin E Franklin

**Affiliations:** 1 Department of Psychiatry and Human Behavior, Brown University School of Medicine, Providence, RI USA; 2 Department of Psychiatry, University of Pennsylvania School of Medicine, Philadelphia, PA USA; 3 Department of Psychiatry and Behavioral Sciences, Duke University Medical Center, Durham, NC USA

## Abstract

This paper presents the rationale, design, and methods of the Pediatric Obsessive-Compulsive Disorder Treatment Study II (POTS II), which investigates two different cognitive-behavior therapy (CBT) augmentation approaches in children and adolescents who have experienced a partial response to pharmacotherapy with a serotonin reuptake inhibitor for OCD. The two CBT approaches test a "single doctor" versus "dual doctor" model of service delivery. A specific goal was to develop and test an easily disseminated protocol whereby child psychiatrists would provide instructions in core CBT procedures recommended for pediatric OCD (e.g., hierarchy development, in vivo exposure homework) during routine medical management of OCD (I-CBT). The conventional "dual doctor" CBT protocol consists of 14 visits over 12 weeks involving: (1) psychoeducation, (2), cognitive training, (3) mapping OCD, and (4) exposure with response prevention (EX/RP). I-CBT is a 7-session version of CBT that does not include imaginal exposure or therapist-assisted EX/RP. In this study, we compared 12 weeks of medication management (MM) provided by a study psychiatrist (MM only) with two types of CBT augmentation: (1) the dual doctor model (MM+CBT); and (2) the single doctor model (MM+I-CBT). The design balanced elements of an efficacy study (e.g., random assignment, independent ratings) with effectiveness research aims (e.g., differences in specific SRI medications, dosages, treatment providers). The study is wrapping up recruitment of 140 youth ages 7–17 with a primary diagnosis of OCD. Independent evaluators (IEs) rated participants at weeks 0,4,8, and 12 during acute treatment and at 3,6, and 12 month follow-up visits.

NCT00074815

## Introduction

The Pediatric Obsessive-Compulsive Disorder Treatment Study Part II (POTS II) evolved out of a collaborative relationship among investigators at the University of Pennsylvania (Drs. Edna Foa & Martin Franklin), Duke University (Dr. John March), and Brown University (Drs. Henrietta Leonard & Jennifer Freeman) and their respective research teams that began years earlier with the Pediatric Obsessive-Compulsive Disorder Treatment Study (POTS I). The POTS I project was the first randomized trial in pediatric obsessive-compulsive disorder (OCD) to compare directly the efficacy of an established medication (sertraline), OCD-specific cognitive behavioral treatment (CBT), and their combination, to a placebo control condition in the initial treatment of children and adolescents with clinically significant OCD [1,2].

The ideal initial treatment for OCD in youth is CBT alone or CBT in combination with a serotonin reuptake inhibitor (SRI) [2-4]. However, despite expert recommendations to start with CBT or CBT plus an SRI, pharmacotherapy with an SRI alone is a widely used initial treatment for OCD in patients of all ages [5,6]. In addition, most patients who receive pharmacotherapy evidence a partial response, with clinically significant residual symptoms [2].

POTS II was designed to investigate two different CBT augmentation protocols in children and adolescents with a diagnosis of OCD who are partial responders to defined adequate SRI pharmacotherapy. Specifically, POTS II is a balanced 3 (site) × 3 (treatment conditions) × 4 (repeated measures) masked randomized parallel group controlled trial that compares 12 weeks of medication management (MM) provided by a study psychiatrist with two types of CBT augmentation: (1) MM + OCD-specific CBT as delivered by a study psychologist (MM+CBT); and (2) MM + instructions in CBT (MM+I-CBT) delivered by the same study psychiatrist who provides MM. The design has elements of an efficacy study (e.g., random assignment, independent ratings, checks on treatment fidelity), but the primary aim of the study was not to test the relative difference between two different psychotherapy approaches, but rather how to implement CBT for pediatric OCD in a format that can be available to the most possible patients.

We hypothesized that a two-doctor model including a highly-trained CBT therapist for OCD would be more efficacious, but perhaps more expensive or otherwise unavailable to many people suffering from OCD. The MM+I-CBT approach, if shown to be comparable overall or with some subset of the patients treated, could be a means where community psychiatrists could be trained in an approach that is efficacious for pediatric OCD, but still feasible within a community practice. While differences between MM+CBT and MM+I-CBT cannot be attributable to the specific treatment components of one treatment versus another (e.g., in-session exposure with response prevention (EX/RP)), our design will allow us to estimate the effect size associated with each specific treatment in a "real world" population of youth with OCD. Additionally, the design allows for an examination of the feasibility of the I-CBT approach (i.e., does this treatment work at all? Do patients attend? Can physicians do CBT in this context?). Finally, the data from this study will begin to answer questions about which treatments may work best for whom (e.g., do those youth with OCD who are more ill require MM+CBT while those who are not as ill may do fine with the less intensive treatment?). This report presents the rationale for the study, describes the design choices made, and outlines the methods used to carry out the trial.

## Background for POTS II

Obsessive Compulsive Disorder (OCD) is a serious and significant psychiatric disorder in early childhood, affecting between 0.5% [7] and 2–3% of children [8,9]. Among adults with OCD, 1/3 to 1/2 develop the disorder in childhood or adolescence [10]. OCD severely impairs academic, social and family functioning [11-14]. Thus, effectively treating OCD in young people may improve functioning and reduce lifelong morbidity, resulting in significant public health benefit.

### Evidence-based Treatment of Pediatric OCD

#### Evidence for SRIs

With respect to SRI treatment of OCD, the pediatric literature is consistent with the adult trials in revealing: (1) little placebo effect; (2) a 30–40% reduction in OCD symptoms, which corresponds to an average 6 point decrease on the Child Yale-Brown Obsessive Compulsive Scale (CY-BOCS); and (3) clinical effects beginning at three weeks, reaching a plateau at after ten weeks [15]. Notably, the majority of patients are left with residual symptoms even after an adequate course of treatment with SRI medication [2].

Experts recommend combining CBT or, less preferably in light of their relative side effect profiles, an atypical neuroleptic to SRI treatment in partial responders [3,4]. To date, no controlled studies have evaluated the efficacy of augmentation treatment in pediatric OCD, which leaves the field without an adequate scientific foundation to guide the management of partial response.

#### Evidence for CBT

Expert clinical panels have long recommended starting with CBT or CBT plus an SRI as the treatment of choice for OCD in youth [3,4], but it is only recently that practice guidelines have been supported by the evidence base [2,16]. The CBT outcome literature in pediatric OCD began with age-downward extension of protocols found efficacious with adults, then publication of single case studies, case series, and open trials involving these protocols [17-20]. These uncontrolled evaluations yielded remarkably similar and encouraging findings across settings and cultures: at post-treatment, the vast majority of patients were responders, with mean CY-BOCS reductions ranging from 50% – 67%. This pilot work set the stage for controlled studies evaluating the efficacy of CBT, one of which was published in the late 1990s [21], four that have been published more recently [2,22-24], and one that has recently been completed (J. Piacentini, personal communication).

Most of the studies of CBT outcome in pediatric OCD have employed similar protocols involving weekly treatment over 12–14 weeks (see references above). In contrast, Weaver and Rey used an intensive CBT protocol that included two information gathering sessions followed by 10 daily sessions of CBT over 2 weeks [20]. Franklin et al. found no differences between 14 weekly sessions over 12 weeks or 18 sessions over 4 weeks, but interpretation of this finding is hampered by the lack of random assignment [17]; a similar but larger study in adults found no difference at follow-up between intensive and twice-weekly CBT [25]. Storch et al. randomized pediatric OCD patients to receive either intensive or weekly CBT and found that patients respond well to CBT delivered either weekly or intensively [23].

The three pediatric CBT pilot studies that have included a follow-up evaluation support the durability of CBT, with therapeutic gains maintained up to 9 months post-treatment [17,18,20]. Moreover, since relapse commonly follows medication discontinuation, the finding of March et al. that improvement persisted in six of nine responders following the withdrawal of medication provides limited support for the hypothesis that CBT inhibits relapse when medications are discontinued [18]. Follow-up data from Barrett et al.'s study further indicate the durability of gains made in CBT for pediatric OCD [26].

A recent meta-analysis of CBT for OCD in pediatric patients found that CBT generally outperformed SRIs alone providing further support to the evidence base for CBT [16]. However, further subanalyses of the data suggest that this finding was confounded because many of the open CBT treatment studies included in the meta-analysis included patients receiving a serotonin reuptake inhibitor (SRI) in conjunction with their CBT treatment [27,28]. Only 4 or 18 CBT studies examined the efficacy of CBT in children on no medication [27]. The literature suggests that a majority of children presenting for CBT treatment for OCD are already receiving stable SRI treatment, indicating a need to clarify the augmenting role of CBT in treatment of OCD.

#### Evidence for combined treatment

POTS I is the only study that has directly compared combined treatment with CBT and SRI pharmacotherapy in a randomized controlled trial. POTS I's findings on the primary continuous outcome measure, the CY-BOCS [29], indicated a statistically significant advantage for CBT alone, sertraline alone, and combined CBT-sertraline treatment relative to placebo. In addition, children receiving combined treatment had a larger reduction in OCD symptoms than CBT or sertraline alone, which did not differ from each other [2]. Rates of clinical remission were: Combined treatment (53.6%; 95% CI 36–70%), CBT (39.3%; 95% CI 24–58), sertraline (21.4%; 95% CI 10–40) and placebo (3.6%; 95% CI 0 – 19). Combined treatment did not differ from CBT (p = .42), but did differ from sertraline (p = .026) and from placebo (p < .001). CBT did not differ from sertraline (p = .24), but did differ from placebo (p = .002), whereas sertraline did not (p = .10). The authors concluded that children and adolescents with obsessive-compulsive disorder should begin treatment with the combination of CBT plus an SRI or CBT alone.

The results of the first POTS study also suggest that delivery of concomitant CBT can help reduce SRI doses. The median dose of SSRI for children receiving combination treatment was 150 mg, as compared to 200 mg for children receiving sertraline alone or placebo [2]. However, SRI partial responders may constitute a different and perhaps more treatment non-responsive sample than those who have participated in studies of initial treatments. If there were evidence that CBT augmentation is effective then further testing about its utility in pharmacotherapy reduction and discontinuation could be pursued in future studies.

### Augmentation of medication

In adults, SRI treatment of OCD has generally been augmented with antipsychotic medications, which cause a significant risk for adverse events [30]. More recently, data have emerged in support of CBT augmentation of SRI treatment in adult OCD [31]. Simpson and colleagues found that 8 weeks of CBT augmentation (17 sessions) was superior to 8 weeks (17 sessions) of augmentation with stress management training, but that 17 sessions of CBT was not enough for most patients to achieve an excellent response. They found that their study participants did not do as well as participants in other studies who received 15 sessions of OCD treatment daily prior to SRI exposure [31].

While these data were not available during the study design phase, in treatment of pediatric OCD, the empirically supported protocol is 14 sessions over 12 weeks [2] and community treatments average approximately 10 sessions [32]. Thus, the decision in the current study was to follow the session timeline found to be efficacious in previous studies of childhood OCD. However, similar results may develop in which children receiving an adequate SRI treatment do not respond as well as children in previous OCD treatment studies. In pediatric OCD, there are no published augmentation trials, nor do we know of any in progress. Three published open studies in the child and adolescent literature suggest incremental benefit when CBT is added to SRI pharmacotherapy in SRI partial responders. One study reported a 50% CY-BOCS reduction following CBT in patients, 2/3rds of whom were partially responsive to an SRI [18]. Similar results (59% CY-BOCS reduction) [17,20] have been reported in youth who were on a variety of SRIs when receiving CBT. Thus, although CBT may be an efficacious augmentation treatment in SRI partial responders, this has not been established in a randomized controlled trial in this patient population against an appropriate control condition. Specifically, there is little information in pediatric OCD about whether CBT is as efficacious an augmentation agent as a first line treatment.

### Availability of CBT

Despite expert recommendation that CBT with a strong emphasis on exposure with response prevention (EX/RP) should be a first-line treatment for OCD in children and adolescents [4], several barriers may limit its widespread use. First, due to low base rates in the community as compared with other anxiety disorders, few therapists have extensive experience with CBT for pediatric OCD [7,33]; thus, CBT typically is available only in areas associated with major medical centers if at all. In our clinical experience at three different anxiety disorders specialty clinics in three diverse areas of the U.S., it is often difficult to find clinicians in the community with specific expertise in CBT for pediatric OCD. Again based on our experiences, those clinicians who do have these skills often have considerably long waiting lists. The scarcity of CBT practitioners is by no means specific to pediatric OCD, but there are a variety of possible explanations including: 1) insufficient exposure during therapists' training to CBT in general and more specifically CBT for OCD [34], 2) the typical psychologist in clinical practice may not see a sufficient number of pediatric OCD patients to develop expertise, 3) resistance among therapists to adapt their preferred approaches to accommodate newer techniques [34], and 4) an increasing focus on medication management because of limited availability of CBT [35].

Second, even when the treatment is available, our experience has shown that some patients and families reject the community based CBT treatment as "too difficult." Once involved in CBT, some patients find the initial distress when confronting feared thoughts and situations while simultaneously refraining from rituals so aversive they drop out of treatment. However, treatment drop out at this stage may be due to insufficient skill on the part of the clinician, as data from POTS I and other CBT for pediatric OCD treatment studies have had fairly low drop out rates [2], even when the treatment has been provided in the community under the supervision of OCD experts [36].

### Rationale For CBT in the context of medication management

Because of the difficulty in obtaining CBT for OCD in the community, it is important to know whether instruction in CBT procedures in the context of medication management by the treating pharmacotherapist could be beneficial for at least some children with residual OCD symptoms despite being on medication. Such instruction may enhance the typical partial response to SRI and be easier to disseminate than the traditional dual doctor model. In addition, instruction in CBT procedures may be more feasible for clinicians operating in settings where in-session therapist-assisted exposure may not be feasible.

A goal of POTS II was to develop an easily disseminated protocol whereby child psychiatrists could instruct patients in CBT procedures comparable to the recommended CBT augmentation strategies. Additionally, by testing a "one-doctor" (instructions in CBT in the context of medication management) versus "two-doctor" (therapist assisted CBT with psychologist in combination with MM by child psychiatrist) model, it may be possible to determine which pediatric OCD patients (i.e., those with more severe illness, those with certain co-morbidities or other external stressors, younger vs. older patients, etc...) most benefit from a full course of more intensive CBT which would allow for a more judicious use of limited resources.

The POTS II study fills gaps in current pediatric OCD research in the following ways: 1) POTS II has many aspects of an effectiveness study. Children are not treatment-naïve and this provides a very different sample from one which examines initial treatment [2]. The inclusion criteria also are broad, to promote generalizability to community practice. 2) POTSII is an augmentation study. All children who entered the study were on a stable dose of an SRI. A version of CBT was added to the treatment regimen, to examine the incremental benefit of adding CBT for children on a stable dose of medication. Because CBT expertise in community settings is limited and because most treated children and adolescents with OCD receive medications, it would be of substantial public health value to know whether a practicable version of CBT that can be delivered by child psychiatrists in the context of medication management can be successful in augmenting the outcome by medication alone for children and adolescents (with OCD) who are partial responders to an SRI. 3) POTSII provides preliminary steps towards dissemination. This study will allow us to answer the question of whether CBT can be adapted to be delivered in shorter and fewer sessions, to allow for potential of increased access and delivery by more providers in the context of other treatment modalities, such as a medication management visit. POTS II will also address the question of whether all patients need therapist-assisted exposure or if some patients improve with instructions alone, as a step toward developing a stages of treatment model for pediatric OCD.

## Specific aims of POTS II

The collaborative R01 grant proposal was funded in 2003 by the National Institute of Mental Health. Following several months of intensive training in study procedures, patient enrollment began in 2004, with anticipated completion of recruitment in January 2009. The specific aims are as follows:

Our primary specific aim for Phase I is:

1. To compare the short-term efficacy of MM+CBT and MM+I-CBT to each other and to MM alone for OCD symptoms and functional impairment for patients who are partial responders to SRIs and seek augmentation treatment.

Our primary specific aim for Phase II is:

2. To compare maintenance of gains monthly for six months on OCD symptoms and functional impairment for patients who responded to MM+CBT and MM+I-CBT after both forms of treatment are discontinued.

Our secondary aim is:

3. To explore predictors of response (Phase I) and relapse (Phase II), including demographics, age of onset, comorbid tics, insight, initial severity, comorbid internalizing and externalizing symptoms, and family psychopathology.

As explained in more detail in the introduction, the design balanced elements of an efficacy study (e.g., random assignment, independent ratings) with effectiveness research aims (e.g., differences in specific SRI medications, dosages, treatment providers).

## Methods of POTS II

The study is currently wrapping up recruitment of a volunteer sample of 140 youth age 7–17 with a diagnosis of OCD based on criteria in the 4^th ^edition of the Diagnostic and Statistical Manual of Mental Disorders (DSM-IV). All children who participated in the study had by definition experienced a partial response to SRI pharmacotherapy. Children were randomly assigned to one of three possible treatment conditions: (1) Medication Management (MM) provided by a study psychiatrist; (2) OCD-specific CBT as delivered by a study psychologist in addition to MM by a study psychiatrist (MM+CBT); and (3) instructions in CBT (MM+I-CBT) delivered by the study psychiatrist assigned to provide MM.

### Alternate design considerations

Although we believe that the experimental design we selected provides a fair and ecologically valid test of two distinct models for providing CBT augmentation to SRI partial responders, several design alternatives and considerations warrant elaboration:

#### Why Not Compare CBT Augmentation to Pharmacotherapy Augmentation?

Augmentation of SRIs with atypical neuroleptics such as risperidone (RIS) is a clinical strategy supported by open trials as well as by one randomized controlled trial, albeit with adult OCD [37]. Although this is an important research question, such a trial was unwarranted at the time the current study was developed due to the lack of evidence for the efficacy and safety of RIS augmentation for children and adolescents.

#### Why Not Compare MM+CBT to I-CBT Conducted by a Psychologist?

Although the reduced visit schedule, contact time and CBT component array that characterize MM+I-CBT amount to a "low dose" version of CBT, the driving rationale for MM+I-CBT is not primarily a test of low versus high dose CBT but rather of a single versus a two doctor model for administering CBT to SRI partial responders. Because we intentionally crossed dose with provider in the MM+I-CBT condition, we did not elect a third study arm in which a low dose version of CBT was administered by a study psychologist. First, the importance to the field of pursuing multiple avenues in which to disseminate CBT ultimately led us to choose the I-CBT program administered by the psychiatrist. Second, given that we still needed to include a control condition, adding this cell to the present design would have reduced power to the point where examination of all of our study's primary aims would have been severely compromised.

#### Why Not Do Double-Blinded SRI Discontinuation in Phase II?

We also considered and ultimately discarded the option of re-randomizing MM+CBT responders to either continue or discontinue SRI. This design would have been a direct test of the need for maintenance medication in CBT augmentation responders. Although this is an interesting and important question in and of itself, it was not possible within the constraints of time and budget. While this type of approach has great appeal because it generates knowledge about the optimal length of treatment, we decided against utilizing an active-maintenance-treatment design, as a host of scientific, methodological, clinical, and financial factors mitigated against such a design [38].

#### Why not a more tightly controlled randomized control trial design?

A primary goal of this study was to take an initial step beyond a traditional efficacy study and broaden the population of children and adolescents with OCD that could benefit from the study. In the spirit of effectiveness research, the sampling frame was designed to recruit a broadly representative sample of moderately to severely ill youth with OCD who are seeking treatment for SRI partial response, while still including efficacy elements, such as randomization and carefully specified in/exclusion criteria, which maximize internal validity (see Table 1, Table 2).

**Table 1:** Inclusion criteria and rationale

*Inclusion Criteria*	*Rationale*
Age 7 – 17 inclusive	Matches developmental sensitivity of treatments and measures
DSM-IV Diagnosis of OCD	Disorder of interest
CY-BOCS total score ≥ 16	Indicates clinically important OCD
Partial responder to optimized SRI trial	Target population of interest
Outpatient	Inpatient care confounds study treatments

**Table 2:** Exclusion criteria and rationale

*Exclusion Criteria*	*Rationale*
Other primary or co-primary psychiatric disorder	May require additional or different treatments
Suicidal ideation with intent	May require additional or different treatments
Pervasive Developmental Disorder(s) (including Asperger's syndrome)	May require additional or different treatments
Thought Disorder	May require additional or different treatments
Concurrent treatment with psychotropic medication (other than stable psychostimulant and/or certain uses of clonidine, tenex, trazodone, or neuroleptic) or psychotherapy outside study	Confounds internal validity of treatment assignment
Prior failed trial of adequate dose of CBT for OCD	Confounds internal validity of treatment assignment; unsystematic sampling bias
PANDAS/maintenance antibiotic for OCD/tics	Confounds internal validity of treatment assignment
Mental Retardation	Would not permit specified CBT treatment
Pregnancy	Potential risk of medication to fetus

### Entry criteria

Subject eligibility for study entry was assessed in a stepwise process, to reduce patient burden and promote efficiency. This step-wise process has been previously described, but consists of a series of assessment "gates," at which subject eligibility is evaluated [1]. The primary entry criteria for the study are subjects who (1) meet DSM-IV criteria for OCD as their primary diagnosis; (2) have evidenced a predefined partial response to an adequate trial of an SRI (as defined below); and (3) still have residual OCD symptoms severe enough to warrant additional treatment, as measured by a score 16 or above on the CY-BOCS (see Table 1).

The choice of a CY-BOCS entry score of 16 was based on two factors: (1) this represents a threshold entry score below which subjects would be excluded in most OCD treatment protocols and (2) an entry CY-BOCS score below this would leave insufficient room for improvement necessary to detect a treatment effect. Entry criteria were determined by assessment. Primary OCD diagnosis was determined from the Anxiety Disorders Interview Schedule [39]. As previously described, OCD severity was determined by the CY-BOCS.

### Inclusion and exclusion criteria

In the spirit of effectiveness research, the sampling frame was designed to recruit a broadly representative sample of youth with OCD seeking augmentation of SRI partial response, while still including key efficacy elements (e.g., randomization, specified inclusion criteria) to ensure internal validity. As described previously, the effectiveness context of this study implied a framework in which we would expect some variability across patients. Our inclusion criteria were purposefully broad to allow for a group of patients who were indeed ill, but also highly representative of a large portion of children and adolescents with OCD who are partial responders to medication treatment.

#### Allowable concurrent psychotropic treatment

To increase generalizability beyond the study, concomitant psychotropic medications were allowed as needed for treatment of common comorbidities (for example, attention-deficit hyperactivity disorder (ADHD), tics, other anxiety disorders, and sleep problems) following cross-site review by the study psychiatrists. To preserve research integrity, potential subjects taking concomitant psychotropic medications in addition to their SRI were reviewed by a cross-site committee before being permitted to enter the study. To provide good clinical care, the physician treating the patient in the study was aware of all medications that the patient was being prescribed and coordinated care with the outside prescriber as needed. All concurrent medications were assessed prior to entrance into the study to ensure that the child was on a stable dose (defined as 4 weeks for ADHD psychostimulants and 12 weeks for other medications) prior to study entry and that the dose did not change during the acute study phase.

#### Allowed concurrent psychosocial treatment

Patients currently receiving supportive psychotherapy, either in individual or family format, were allowed to continue as long as the following conditions were met: (1) The patient was in this treatment for 4 months or more; (2) The supportive treatment was at a stable frequency not to exceed once per week; and (3) The treatment did not include cognitive-behavioral therapy for OCD.

#### Prior failed trials of CBT

Prior exposure to CBT treatment, per se, was NOT an exclusion criterion except if the child received an adequate dose of CBT, which was defined as at least 10 sessions of CBT that included use of a symptom hierarchy and therapist-assisted exposure/response prevention. Assessment of an adequate dose of CBT included a review with the child and parent of the content and homework of previous CBT for OCD and, when possible, a review with the treatment provider regarding the techniques included in the previous treatment. All decisions regarding inclusion or exclusion from the study were made by the cross-site panel.

#### SRI medication treatment

To ensure maximum generalizability, eligible SRI medications were determined by expert recommendations and standard treatment of OCD in the community (see Table 3). Citalopram, escitalopram, fluoxetine, fluvoxamine, paroxetine, paroxetine-controlled release, and sertraline were included as eligible SRI medications both because of their common use in treatment of OCD in children and research evidence supporting their efficacy in reducing OCD symptoms [15,40-43]. Although not typically first-line medication treatments of OCD, clomipramine, venlafaxine, and venlafaxine-extended release are prescribed after a patient fails a trial of an SRI [21,44-46]. Because this study targeted partial responders of medication who may have been partial responders to multiple medication trials, these medications were also included as allowed SRI medications.

**Table 3:** SRI dosing

Drug	Usual Starting dose	~ Mean Dose*	Upper Dose	Incremental Dose
Citalopram**	20	40	60	20

Clomipramine	50	150	250	50

Escitalopram**	10	20	30	10

Fluoxetine	20	40	60	20

Fluvoxamine	50	175	250	50

Paroxetine	20	30	50	10

Paroxetine-CR	20	30	50	10

Sertraline	50	125	200	50

Venlafaxine**	25	100	225	25

Venlafaxine XR**	37.5	112.5	225	37.5

*Mean dose derived from registration trials, expert recommendation and the applicant's clinical experience

**Not included in Expert Consensus Guidelines

### Identification of partial responders

To meet the definition of partial response, patients must have had at least three weeks of stable OCD symptoms at an SRI dose that is equal to the upper dose (Table 3) OR patients must have experienced adverse effects as a result of dosage increase OR patients must have shown a flat dose-response curve for one dose increment above the minimum expected starting dose (Table 3). Because most patients who respond to a SRI do so at a mean dose considerably lower than the maximum, an aggressive forced titration strategy to raise the dose to the "maximum tolerated therapeutic dose" independent of response status was deemed unwarranted clinically, as it could impose an undue experimental and adverse event burden on patients. On the other hand, the possibility of suboptimal dosing could not be unthinkingly discounted since some patients do respond when the dose is raised to the maximum. To balance these imperatives – maximizing benefit of SRI, minimizing the risk of high dose SRI, and minimizing unnecessary delay in implementing augmenting treatment, a "within subject" definition of "adequate dose" that included both dose-response and time-response considerations was implemented.

#### Persistent symptoms define partial response

While one standard definition of adequate clinical response is a 25–30% decline in symptomatology, most experts agree that the persistence of significant OCD symptomatology in the face of adequate treatment would also qualify as an inadequate response to treatment. There are three reasons we defined partial response on the basis of persistent OCD, rather than by a pre-defined CY-BOCS symptom reduction:

First, many psychiatric patients may receive their initial treatment in primary care, and then be referred for psychiatric consultation. In these cases, obtaining a CY-BOCS change score would not be feasible, making this standard unobtainable.

Second, based on the mean doses in industry funded trials (each of which used forced upward titration schedules) and the POTS I study, after 12 weeks of adequate treatment, full remission is unlikely with a higher SRI dose or longer treatment duration even if such increases were possible, which is typically not the case.

Third, upward titration is often limited by adverse events, which may or may not be persistent.

**Figure 1:**
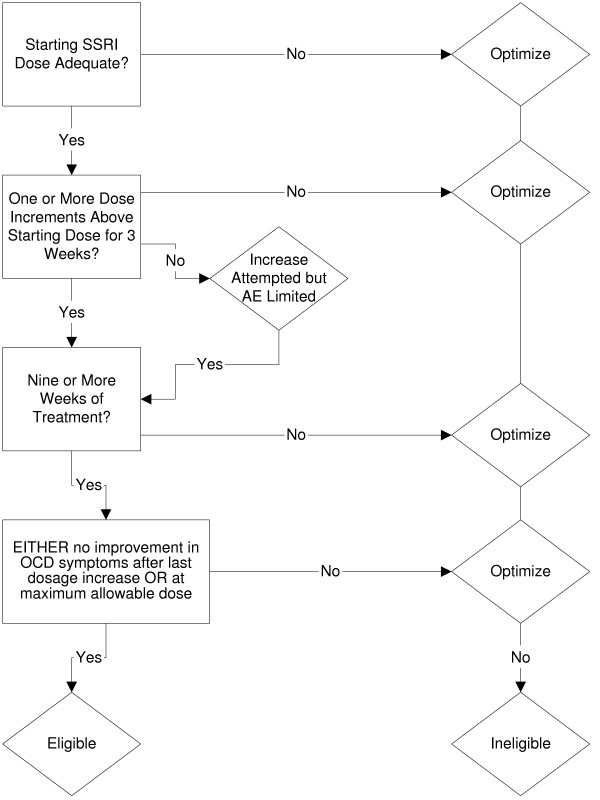
**Flow chart for partial response**.

To establish partial response, a POTS II pharmacotherapist considered the following (see Figure 1): 1) Adequate trial at or above the minimum starting dose; 2) Maximum dose; 3) Intolerable side effects at a dose above his or her current dose; 4) Stable current dose for 3 weeks; 5) Minimum of 9 weeks of treatment.

If the patient had been treated with an SRI for at least nine weeks AND had been at a stable dose for the past three weeks, e.g., the dose response curve was flat indicating no further improvement in OCD symptoms, OR the patient did not tolerate a dose increase to the next higher dose OR the patient had been at the maximum allowable dose for three weeks, then the patient was eligible for randomization to one of the three POTS II treatment conditions. Patients not meeting this definition when presenting initially for study participation were allowed to return for reevaluation by the study team when sufficient dose and duration criteria had been met to be considered for study entry. At that point, if eligible, the patient was promoted to randomization.

### Waiver of optimization

Patients who had specific circumstances that precluded the use of the above medication optimization paradigm were able to receive review by a cross-site committee of study psychiatrists to determine whether they should be considered effectively optimized. This included situations in which the patient had adverse events on another SRI medication and/or parent or psychiatrist reluctance or refusal to raise the SRI dose. Waivers were documented and coded for later consideration in data analysis.

Food and Drug Administration (FDA) advisories recommend that health care providers carefully monitor patients receiving antidepressants for possible worsening of depression or suicidality, especially at the beginning of therapy or when the dose either increases or decreases. Subsequent to the FDA's issuance of the "black box" warning for SRI medication, concern of adverse effects at increasingly higher doses of medication increased among parents, pediatricians, psychiatrists, and study personnel. Thus, patients who had not achieved optimization criteria (e.g., they had not experienced a flat dose-response curve at a moderate to low level of medication), but whose parents or provider declined further increases to the medication were allowed study entry via the previously described waiver process.

### Assessment

The primary instrument for assessing OCD was the CY-BOCS, which assesses obsessions and compulsions separately on time consumed, distress, interference, degree of resistance, and control [47,48]. We used the CY-BOCS symptom checklist and severity scale to inventory past and present OCD symptoms, initial severity, total OCD severity, relative preponderance of obsessions and compulsions, and degree of insight [29]. The CY-BOCS is a clinician-rated instrument that involves merging data from clinical observation and parent and child report; independent evaluator (IE) reliability training is described below.

Several procedures were set in place to maintain the rater's blind to patient treatment status. The IEs at all sites were doctoral level psychologists with specific training in the assessment of pediatric OCD. Notably, the IEs at each site were not actively involved in research program beyond their role as IE, thus further promoting their independence. They did not attend weekly study coordination or treatment supervision meetings or weekly cross-site calls, however, there was a monthly cross-site phone meeting for the IEs only led by the IE coordinator from the Duke site. IEs rated patients on the day of treatment, but in a different physical location to ensure rater blinding to treatment status. Patients were instructed not to disclose treatment status to their IE; the IE was also instructed not to inquire about treatment status. To assess the adequacy of the IE blinding procedure, IE were asked to guess assignment to condition (MM+CBT, MM+I-CBT, or MM) at the end of the treatment.

Improvement and severity ratings were obtained from the therapist (MM+CBT) and psychiatrist (MM+ and MM+I-CBT groups) at every 4th treatment visit. All self- and parent-report measures were completed on scheduled visit days. In the event that a patient/parent was unable to read the self-report measures, personnel unconnected with the study provided assistance. Because all study participants received active SRI treatment, clinical assessment of side effects using the child, parent, and clinician versions of side effects and suicidal and homical ideation was completed by the physician for all subjects in the study irrespective of treatment assignment. The pharmacotherapist assessed side effects and possible risk factors associated with SRIs; thereby tracking adverse occurrences between study visits, the severity, possible causes and outcome. Additional measures were included in the study to address secondary aims and questions of predictors of treatment response (see Table 4).

**Table 4:** Measures By domain, variable type and rater

*MEASURE*	*Domain*	*Who*	*Gates*	*Baseline*	*Acute Treatment*	*Naturalistic Follow-up*
Phone screen	In/Exclusion	SC	X			
Demographics, history	Caseness	SC	X			
Treatment history	Caseness	T	X			
ADIS	Caseness/Comorbidity	T	X			
Yale Global Tic Scale	Tic disorders	T	X			
PANDAS interview	PANDAS	T	X			
CY-BOCS	OCD	T, IE	X	X (IE)	X	X
NIMH Global (Impairment)	OCD	T, IE	X	X (IE)	X	X
Clinical Global (CGI-I and CGI-S)	OCD severity	T, IE	X	X	X	X
COIS	Functional impairment	C, P		X	X	X
Expectancy Ratings – Medication	"Non-specific" effects	C, P, T		X	X	X
Expectancy Ratings – Psychotherapy	"Non-specific" effects	C, P, T		X	X	X
Consumer satisfaction	Consumer satisfaction	C, P		X	X	X
IE Blindness	IE Blind	IE			X	X
MASC	Child anxiety	C		X	X	X
CDI	Child depression	C		X	X	X
Conners Parent Rating Scale	Disruptive behaviors	P		X	X	X
BSI	Parent psychopathology	P		X	X	X
Family Assessment Measure	Family functioning	P		X	X	X
PQ-LES-Q	Quality of life	P		X	X	X
CGAS	Quality of Life	T		X	X	X
HARM form	Adverse Events: Suicidal and homicidal ideation and behavior	T	X	X	X	X

Pediatric Adverse Events Rating Scale (PAERS)	Adverse Events	C, P, T	X	X	X	X

Teasing Questionnaire (TQ)	Social Functioning	C		X		

SEQ-S	Social Functioning	C		X		

Attitudes Toward My Child	Family Functioning	P		X		

Parent Reaction Questionnaire	Family Functioning	P		X		

WAM	Child Emotionality	C		X		

SC = study coordinator; T = clinician rated, C = child self-report, IE = independent evaluator rated, P = Parent rated self-report

### Three treatment conditions

#### MM

Once randomized, all patients were assigned to a child/adolescent psychiatrist from whom they received maintenance SRI medications (MM) for the duration of the study. MM visits were conducted on a maintenance visit schedule at weeks 1, 2, 4, 6, 8, 10, and 12. In accordance with sound clinical practice, in addition to monitoring clinical status and medication effects, pharmacotherapists offered general encouragement to resist OCD and told patients that medication will make this easier. In distinction to the pharmacotherapist in the MM+I-CBT assignment who implemented a systematic EX/RP protocol, the pharmacotherapist in MM alone and MM+CBT implemented no systematic or unsystematic cognitive therapy (CT) or EX/RP program. Insight-oriented or interpersonal psychotherapy, other CBT interventions, or family therapy provided by the study psychiatrist were similarly proscribed during the 12-week study period.

#### I-CBT

MM+I-CBT is a protocol in which the psychiatrist who manages medication also provides instructions in the CBT procedures that have been found to help reduce OCD symptoms, namely EX/RP. MM+I-CBT was constructed as a single-doctor "best practice" treatment with three primary goals in mind: (1) inclusion of the main psychoeducational and EX/RP components of the full CBT protocol; (2) feasibility of training psychiatrists to perform the CBT component of MM+I-CBT; (3) integration with protocol medication management visits; and (4) feasibility of implementation with the constraints of a busy practice oriented primarily toward pharmacotherapy. As shown in Table 5, MM in MM+I-CBT were administered according to the MM protocol (7 visits over 12 weeks), with additional time for I-CBT provided via: (1) increasing the time available for I-CBT by increasing visit length; and (2) by emphasizing I-CBT at each session, which was permissible and practical because the time demands of maintenance pharmacotherapy are minimal relative to acute titration visits. Fewer or shorter sessions would have vitiated the "best practice" philosophy of MM+I-CBT; given that we hypothesized an intermediate response rate for MM+I-CBT, more or longer sessions would have vitiated the comparison to MM+CBT and would be unfeasible in clinical practice.

**Table 5:** I-CBT treatment protocol

*Wk/Visit Number*	*Time (Min)*	*Goals*
Week 1/Visit 1	90	Psychoeducation
Week 2/Visit 2	50	Mapping OCD, EX/RP
Week 3-phone	10–15	Ckeck-in for exposure
Weeks 4, 6, 8/Visits 3, 4, & 5	30	EX/RP
Week 5-phone	10–15	Check-in for exposure
Week 10/Visit 6	30	EX/RP Relapse prevention
Week 12/Visit 7	30	End of treatment

MM+I-CBT does not include the following components that are part of the full CBT protocol: (1) Cognitive Training (CT) except for bossing back metaphors, and externalizing techniques, such as using a "nickname" for OCD; (2) the fear thermometer as an aid to creating and re-evaluating the stimulus hierarchy; (3) detailed hierarchies addressing different aspects of OCD; (4) imaginal exposure instructions; (5) therapist-assisted EX/RP in the office; (6) dyadic parent sessions except as noted; (7) detailed instructions regarding pitfalls in CBT and methods for moving stalled treatment forward. Exclusion of these components, while not detracting from the core components of CBT, was necessitated by both the time and the expertise required for their implementation [49].

#### CBT

As Table 6 shows, the CBT protocol to be administered by the study psychologist in the context of MM+CBT consists of 14 visits over 12 weeks involving: (1) psychoeducation, (2), CT, (3) mapping OCD, and (4) EX/RP. The CBT Treatment Manual used in the study is adapted from March and Mulle [50] and was used in a previous collaborative study of treatments for pediatric OCD [2]. Except for weeks 1 and 2, when patients came twice weekly, all visits were administered on a once/week basis, last one hour, and also include one between-visit 10 minute telephone contact scheduled for weeks 3–12. Psychoeducation, defining OCD as the identified problem, cognitive training, and development of a stimulus hierarchy (mapping OCD) took place during visits 1–4; EX/RP comprised visits 5–12, with the last two sessions incorporating generalization training and relapse prevention. Each session included a statement of goals; review of the previous week; provision of new information; therapist-assisted practice; homework for the coming week; and monitoring procedures. Consistent with sound clinical practice and by virtue of the study design, the psychiatrist and psychologist were aware that the child assigned to MM+CBT was seeing another treatment provider, and conferred regularly as to the clinical progress made by the child. However, there were no treatment dependencies, e.g. the dose of medication did not vary as a function of progress in CBT.

**Table 6:** CBT treatment protocol

*Wk/Visit Number*	*Time (min)*	*Goals*
Week 1/visits 1 & 2	120 (2 visits)	Psychoeducation Cognitive training
Week 2/Visits 3 & 4	120 (2 visits)	Mapping OCD Cognitive training
Week 3–12/Visits 5–14	60 (1 visit/week)	Exposure and response prevention
Visits 11–12	60 (1 visit/week)	Relapse prevention
Visits 1,7 & 11	Included in above-described session time	Parent sessions

All study CBT therapists underwent in-person group and individual training (with study PIs) that included familiarization with the treatment manuals, followed by extensive role-playing of treatment procedures as well as videotaping. To maintain treatment fidelity throughout the study, all sessions were videotaped, all therapists received ongoing, weekly case supervision, and 15% of all CBT tapes were selected at random for review by the CBT supervisor. Supervision consisted of a weekly cross-site conference call in which all actives cases were discussed, review of videotapes of therapy sessions, and feedback to therapists based on tape review. To establish treatment fidelity, two evaluators, trained until they reached at least 80% agreement, reviewed 20% of MM+CBT and 20% of MM+I-CBT tapes randomly selected, ensuring equal representation of each session to ensure that intervention procedures were followed and that no proscribed treatment techniques are being used. Reliability was calculated between the two evaluators as well as between each evaluator and the study therapist. Eighty percent agreement was considered acceptable.

In both the CBT and I-CBT protocols, individualization and developmental appropriateness of treatment was promoted by allowing flexibility within the constraints of fixed session goals. More specifically, therapists adjusted the level of discourse to the specific interests, level of cognitive functioning, social maturity and capacity for sustained attention of each patient. For example, younger patients required more redirection and activities in order to sustain attention and motivation. Adolescents were more sensitive to the effects of OCD on peer interactions, which in turn required more discussion. In addition, cognitive interventions required adjustment to the developmental level of the patient, with adolescents, for example, less likely to appreciate giving OCD a "nasty nickname" than younger children. Developmentally appropriate metaphors relevant to the child's areas of interest and knowledge were also used to promote active involvement in the treatment process. For instance, an adolescent male football player treated with CBT was better able to grasp treatment concepts by casting them in terms of offensive and defensive strategies employed during football games (e.g., handling blitz assignments on the offensive line). Patients whose OCD symptoms entangled family members required more attention to family involvement in treatment planning and implementation than those without as much family involvement. Nonetheless, the general format and goals of the treatment sessions were the same for all children.

### End of treatment recommendations

With the exception of subjects who withdrew consent for treatment and assessment (drop outs) for whom we could not provide recommendations, as the treatment relationship was severed by the patient, ethical principles require that all participants be given recommendations for any indicated further treatment and appropriate referrals at the end acute of acute treatment.

*Patients who completed MM only*, were offered the full course of CBT at no cost from the study team or, if they preferred, were offered community referral(s). The cost of medication management and SRI medication was not provided in the open treatment. MM excellent responders (CY-BOCS ≤ 10) who did not choose open CBT were offered open CBT treatment (at no cost) if they relapsed within six months of ending acute treatment. Based on evaluation of clinical response using a clinician assigned CY-BOCS, excellent responders (CY-BOCS ≤ 10) to MM+CBT and MM+I-CBT received end-of-treatment recommendations based on their clinical status.

*MM+I-CBT non-responders *or partial responders (CY-BOCS > 10) were offered the full CBT protocol at no cost from the study team or, if they prefer, are offered community referral. The cost of medication management and SRI medication was not provided in open CBT treatment.

*MM+CBT non-responders *were referred to community care with specific end-of-treatment recommendations.

Referrals to treatment in the community were made from a standard referral list of providers with expertise in the care of pediatric OCD. To minimize site differences, the process was standardized across the three sites by using a debriefing script. Briefly, this script a) provided the family a chance to state any concerns or questions they had; b) provided a summary of progress, using clinical indicators; c) outlined the possible available treatment strategies, emphasizing those in the assigned treatment arm but also explaining the others; and d) made recommendations about appropriate continued treatment.

### Adjunctive Services and Attrition Prevention (ASAP)

In a long-term trial, ethical and practical obligations require a mechanism for providing services to children and families that are not easily accounted for within the framework of protocol treatments [51,52]. For example, an investigator may find it necessary to address a significant decline in functioning (as opposed to a lack of improvement), as in the case of a severe adverse reaction to medication or dangerousness to self or others. Regardless of the child's assigned treatment condition, children/families who experienced clinical worsening were provided within protocol adjunctive services as follows: two Adjunctive Services and Attrition Prevention (ASAP) sessions were available during acute treatment to provide supportive evaluation services to determine whether the child may remain in the study or other treatment was needed. ASAP sessions were used to prevent premature termination (investigator initiated protocol violation) and dropping out (withdrawal of consent). Sessions were provided by the child's study treatment provider (psychiatrist for MM and MM+I-CBT, psychologist for MM+CBT), and may have been carried out with the child's family. All ASAP procedures were reviewed/approved and documented by a cross-site ASAP panel. ASAP procedures, which provide for optimal sample maintenance and for ethical treatment, have been used successfully in previous multi-site trials [2,51,52]. From a data analytic standpoint, premature termination will be handled via the use of random regression (RR) models which permit estimation of changes in continuous repeated measures on both a population and subject-specific level, without necessitating last observation carried forward (LOCF) or exclusion of subjects with missing data in the dependent variable.

### Sample size and randomization

The total "n" and the group sample sizes were driven by power requirements. Using a conventional definition of responder as those who achieve at least a 30% reduction on the CY-BOCS; we estimated response rates of 70%, 40%, and 10% for MM+CBT, MM+I-CBT, and MM, respectively. The 30% difference in response rates is defined as a difference in the probability of normalization (CY-BOCS less than or = to 10, not a change score of 30%) as the threshold of clinically meaningful difference for purposes of power calculations. Based on data from POTS I, we estimated a modest response for MM alone (10%) and added 30% to that to come up with an expected 40% response rate for ICBT and added another 30% to come up with the response rate for CBT.

Accordingly, a two group X2 test with a 0.050 two-sided significance level would have 80% power to detect the difference between a Group 1 proportion of 0.600 and a Group 2 proportion of 0.300 (odds ratio of 0.286) when the sample size in each group was 42 and the total sample size was approximately 130. To allow for 15% drop outs in intent-to-treat analyses in which all subjects' data are included, we elected a sample size of 150, and expect to complete the trial at a total "n" of approximately 140 subjects. The expected drop out rates were based on data from POTS I in which only 15 of 112 patients (13%) dropped out [2]. Given that we will be using more powerful mixed effect regression models for scalar outcomes and generalized estimating equations for categorical outcomes, power should be sufficient to detect a clinically meaningful effect if one is present.

Using a list created prior to study initiation, randomization was done at the subject level at each site. The actual procedure, which was monitored by the Data Center at Duke, was a stratified randomization on site alone, with randomized permuted blocking (on age, gender, and severity of clinical presentation) within each stratum [53]. Subjects were considered randomized when they learned of their assignment to condition after the baseline assessment. Subjects who dropped out prior to randomization will not be included in data analyses.

### Data management and analysis

Our overall data analytic strategy has been designed to meet the objectives outlined in our specific aims. Accordingly, the data analysis plan consists of: (1) data inspection, (2) descriptive analyses, (3) hypothesis testing, and (4) model specification. Following completion of data collection, analyses will proceed in two steps using a mixed effect "random regression" (RR) model. RR permit modeling of outcomes at the individual level using both individual and cluster level variables while adjusting for the intraclass correlation present in the data [54]. The models do not make assumptions requiring equal sample sizes, and thus covariates, either time varying or time-stationary, can be included. RR also permits estimation of changes in continuous repeated measures on both a population and subject-specific level, without necessitating last observation carried forward (LOCF) or exclusion of subjects with missing data in the dependent variable. Thus, RR provides an efficient and typically more powerful means of assessing time-invariant characteristics (e.g., gender) and time-dependent variables (e.g., treatment dose) on mean changes in the dependent variable [55].

In step one, as recommended by Lavori et al. [56], initial analyses will be conducted under an "intent-to-treat" model in which all assessment points at all visits will be obtained and data analyzed according to treatment assignments at randomization. In step two, we will conduct "completer analyses" on those children completing a full course of treatment. Once past intent-to-treat (ITT) main outcome analyses as advocated by clinical trials experts [53,56], subsequent analyses will focus on predictors of outcome [57,58]. We have sufficient power to detect moderate to large treatment-covariate interactions, but did not stratify on the basis of moderator variables and because the study was not "powered" to include detection of moderator or mediator effects, these analyses are considered secondary to the overall ITT analyses [59]. Numbers needed to treat (NNT) will be used to analyze group differences in remission rates [60]. The NNT represents the number of patients who would need to be treated with CBT to produce one additional responder (i.e., CY-BOCS reduction 30%) beyond that obtainable with I-CBT. Lower NNT scores are better, with most effective therapies for psychiatric disorders showing NNT indices of 3 to 6. Treatment response will be defined in two ways: 1) the primary continuous outcome measure is the IE-rated CY-BOCS and 2) the primary categorical outcome measure is excellent response on the IE CY-BOCS (≤ 10).

Given the intensive fidelity/reliability checks built into the protocol, site differences that move in the same direction – i.e., that differ in quantity or magnitude but not quality or direction of effect – will be seen as contributing to the generalizability of the findings. While Lavori and others have noted that site differences need not be accounted for statistically unless divergent by treatment and clinically meaningful [53,56], site will be accounted for as a fixed effect variable in all analyses.

## Conclusion – potential implications of study

To date, no controlled studies have evaluated the efficacy of any augmentation treatment in pediatric OCD, which leaves the field without an adequate scientific foundation to guide the management of partial response. Finding expertise in CBT for OCD in community settings has proven difficult, and thus it is imperative to determine whether CBT can be effectively disseminated to the mental health providers who most often encounter OCD in their clinical practice, namely child psychiatrists and primary care physicians.

One potential weakness of the study is the cross-cultural relevance of the results. Partial response to SRI medication may be a clinical problem that occurs more frequently in the United States, given overall lower rates of psychopharmacological treatment of pediatric patients outside the United States [61,62]. While the problem of partial response is necessarily less relevant in a culture in which medication is prescribed less frequently, the phenomena remains and this study will provide a better understanding of it.

This study begins to address the effectiveness of CBT using real-world patients, who have had some response to medication but continue to experience clinical levels of OCD symptoms. It compares standard EX/RP for OCD with a briefer, instructional CBT model. As such, the results of this study can provide directions for dissemination of CBT models, suggesting what adaptations should be made to treatment to increase feasibility in real-world settings. In addition, the study may answer initial questions regarding which patients need what treatment, providing empirical support for a stages of care treatment model.

## Competing interests

JSM is a consultant or scientific advisor for Eli Lilly, Pfizer, Wyeth and GlaxoSmithKline. He has equity holdings in MedAvante. He is the author of the Multidimensional Anxiety Scale for Children (MASC) for which he receives royalties, and is a member of a DSMB overseeing research conducted by Astra-Zeneca or Johnson & Johnson. Under separate independent grants, he receives study drug from Eli Lilly and Pfizer for two NIMH-funded clinical trials.

The remaining authors declare that they have no competing interests.

## Authors' contributions

JF, AG, JM, MF, and EF participated in the design and supervision of the study, and helped to draft the manuscript. MS, PM, JS, and MK participated in the implementation and coordination of the study and helped to draft the manuscript. All authors read and approved the final manuscript.
